# Effect of Aging on Superelastic Response in [001]-Oriented Single Crystals of FeNiCoAlTiNb Shape-Memory Alloys

**DOI:** 10.3390/ma18122842

**Published:** 2025-06-16

**Authors:** Li-Wei Tseng, Wei-Cheng Chen

**Affiliations:** Department of Mechatronics Engineering, National Changhua University of Education, Changhua 50007, Taiwan; d1151002@gm.ncue.edu.tw

**Keywords:** shape-memory material, FeNiCoAlTiNb, microstructure, superelasticity, precipitates

## Abstract

In this study, the effect of aging heat treatment on the superelastic properties and microstructure of [001]-oriented Fe_41_Ni_28_Co_17_Al_11.5_Ti_1.25_Nb_1.25_ (at.%) single crystals was investigated using the cyclic superelastic strain test and a transmission electron microscope (TEM). The TEM results reveal that the average precipitate size is around 3–5 nm in the 600 °C/24 h samples, 6–8 nm in the 600 °C/48 h samples, and 10–12 nm in the 600 °C/72 h samples. The results indicate that precipitate size increases as aging time increases from 24 to 72 h. EDS analysis results show decreased Fe and increased Ni when the analyzed line crosses the precipitate region. The diffraction pattern results show that the precipitate has an L1_2_ crystal structure. The thermo-magnetization curves of single crystals under the three aging conditions (600 °C/24 h, 600 °C/48 h, and 600 °C/72 h) show that the values of the transformation temperatures increased from 24 to 72 h. Magnetization was saturated at 140 emu/g under the magnetic field of 7 Tesla. When increasing the magnetic field from 0.05 to 7 Tesla, the transformation temperatures rose. The results indicate that magnetic fields can activate martensitic transformation. From the results of the superelasticity test at room temperature, [001]-oriented FeNiCoAlTiNb single crystals aged at 600 °C for 24, 48, and 72 h presented recoverable strains of 3%, 5.1%, and 2.6%, respectively. Digital image correlation (DIC) results of the aged samples show that two martensite variants were activated during the superelasticity test. The two variants form corresponding variant pairs (CVPs) and improve the recoverable strain of superelasticity. Although maximum recoverable strain was obtained for the 600 °C/48 h samples, the samples show poor cyclic stability at room temperature after applying the 6% strain. According to the DIC results, the retained martensite, which is pinned by dislocations, was observed after the test. The irrecoverable strain was attributed to the residual martensite. For the 600 °C/72 h samples, the large size of the precipitates poses an obstacle to dislocation transformation and formation. The dislocations increase the stress hysteresis width and stabilize the martensite, causing poor recoverability.

## 1. Introduction

Shape-memory alloys (SMAs) show functional properties, such as the superelastic effect (SE) and shape-memory effect (SME) [1,2,3,4]. SE is defined as SMAs that can recover their original shape after removing the stress, and SME is defined as SMAs that can remember their original shape after heating or cooling [1,2,3,4]. Fe-based SMAs are promising with their low manufacturing and material costs and have great potential in a wide range of industrial applications [5]. Most Fe-based SMAs show small recoverable strain (<1%) [6,7,8]. In 2010, Fe_40.95_Ni_28_Co_17_Al_11.5_Ta_2.5_B_0.05_ (at.%) was discovered by Tanaka et al. [9], showing reversible strain almost up to 13% at room temperature. Later, studies on other similar systems, namely, Fe_41.95_Ni_28_Co_17_Al_10.5_Nb_2.5_B_0.05_ and Fe_42.45_Ni_30_Co_15_Al_10_Ti_2.5_B_0.05_, reported a 4% recoverable strain at room temperature [10,11]. For FeNiCoAlXB (X: Ti, Nb, Ta) systems, the transformation system (austenite to martensite) of FeNiCoAlTaB is from face-centered cubic (fcc) to body-centered tetragonal (bct) [9,10,11]. However, a significant reversible superelasticity strain only appeared if the material possessed strong [001] texture, large grain sizes, and coherent precipitates. To gain better insights into the orientation effect, many research teams have focused on FeNiCoAl-based single-crystal systems to understand the orientation of the SE and SME properties without considering the grain size effect [9,10,11].

Ma et al. [12,13] first investigated [001]-oriented Fe_41_Ni_28_Co_17_Al_11.5_Ta_2.5_ (at.%) single crystals without adding boron inside the FeNiCoAl-based systems. The single crystals aged at 600 °C for 90 h showed a 3.75% shape-memory recoverable strain under tension and 2% under compression. The coherent precipitate size was 5 nm, and the precipitate volume fraction was 38%. Evirgen et al. [14] investigated the aging effect on the microstructure and superelasticity of [001]-oriented FeNiCoAlTa single crystal in two aging conditions (600 °C—90 h and 700 °C—7h). The precipitate size of the crystal subjected to the 700 °C aging temperature (3−4 nm) is smaller than that of the crystal subjected to the 600 °C aging temperature. From the tensile test results, the recoverable strain of superelasticity is 3.4% at 25 °C for an aging temperature of 700 °C and 3.4% at 0 °C for an aging temperature of 600 °C. The superelastic functional stability of a [001]-oriented FeNiCoAlTa single crystal aged at 700 °C for 1 h was first characterized by Krooß et al. [15]. The FeNiCoAlTa single crystal shows the cyclic functional degradation behavior. The critical stress is 740 MPa in the first cycle, and the value decreases to 150 MPa in the 100th cycle. The recoverable strain up to 4% is in the first cycle and reduces to a 2.6% recoverable strain in the 100th cycle. The pronounced cyclic degradation is due to dislocation generation at both the martensite–austenite and martensite–precipitate boundaries. The dislocations pinned the martensite phases to inhibit the reverse transformation in austenite, contributing to poor functional stability.

Czerny et al. [16,17,18] investigated the effect of aging temperatures and times on the precipitate morphology and superelastic compression behavior in a [001]-oriented FeNiCoAlTaB single crystal. The average diameter of precipitates is about 3 nm for single crystals (700 °C—0.5 h). The single crystals show the 14.3% recoverable strain compression at −196 °C by comparing the microstructure and superelastic results of both [001]-oriented FeNiCoAlTa and FeNiCoAlTaB single crystals. Adding boron to the single crystals will (1) reduce the average precipitate size and volume fraction, (2) delay β-phase formation, and (3) improve the recoverable strain values [18,19].

Adding Ta will increase the aging times to form the coherent precipitates. Tseng et al. [20] reported that replacing Ta with Ti effectively reduces the aging treatment times for precipitate formation. Based on the [001]-oriented FeNiCoAlTi results, the single crystals with the aging condition (600 °C—24 h) show 6% superelastic strain in tension and 2% superelastic strain in compression at −80 °C. The precipitate’s diameter was measured to be approximately 5 nm. For the aging condition (600 °C—24 h), the maximum recoverable strain is 4.8% in tension, and the precipitate’s diameter is approximately 8−10 nm [21]. Abuzaid et al. [22] investigated the functional fatigue life of [001]-oriented FeNiCoAlTi single crystals (600 °C−3 h). Superelastic property degradation was related to the gradual buildup of local irrecoverable strains, a process induced by plasticity at the martensite–austenite boundary, which acts to pin the interface and prevent reverse transformation. Chumlyakov et al. [23] investigated the two steps of aging (4 h + 2 h) on [011]-oriented FeNiCoAlTi single-crystal particles. When aging at the second step for 2 h, superelasticity and shape-memory effect are 3% and 4.5%, respectively, and after 4 h of aging, the crystals become brittle and fail before applying the 1% strain. Lehnert et al. [24] investigated the superelasticity of [011]- and [123]-oriented micropillar compression FeNiCoAlTi single crystals (650 °C—8 h). Both orientation samples show low reversible strain due to dislocation formation. The two different martensite variants cannot easily accommodate the detwinning of the twinned martensite in both the [011] and [123] orientations. The dislocation movement becomes strong when the detwinning martensite is activated during the superelasticity test. As a result, small recoverable and large irrecoverable strains are found in both orientation samples due to detwinning martensite formation [24].

Adding Nb in FeNiCoAl-based systems can strengthen the austenite matrix. Karaca et al. [25] investigate the superelastic responses in both tension and compression [001]-oriented FeNiCoAlNb single crystals (700 °C—3 h). The aged sample with precipitates of 5 nm in diameter shows 4.5% recoverable strain in tension at −196 °C and 8.8% recoverable strain in compression at −130 °C. Chumlyakov et al. [26,27] characterized the aged [001]-oriented FeNiCoAlNb single crystals (700 °C—0.5 h) with a large reversible tensile strain of 15.3% at −196 °C. According to the constant strain test results of different temperatures, the Clausius–Clapeyron slope is 3.1 MPa/°C [28].

Lauhoff et al. [29] investigated the superelastic properties of [001]-oriented FeNiCoAlTiNb single crystals under different aging temperatures and times. The optimal aging condition of FeNiCoAlTiNb aged at 650 °C for 6 h shows 4% tensile recoverable strain at test temperature −130 °C. The Clausius–Clapeyron slope is around 2.8 MPa/°C between −130 °C and room temperature. However, cyclic superelasticity degradation was observed in the FeNiCoAlTiNb single crystals. The poor functional stability is due to dislocation arrangements. The dislocations appeared at the austenite–martensite boundaries during each cyclic superelasticity test. These dislocations can stabilize the martensite and increase the martensite in each cyclic test, causing large irrecoverable strain.

In summary, the precipitate not only changes the transformation temperatures but also tailors the shape memory and superelastic properties. There are no previous reports on how aging heat treatment affects the precipitate size and the superelastic responses of FeNiCoAlTiNb single crystals. Therefore, this study aimed to investigate the effect of aging on precipitate morphology, martensitic transformation behavior, and superelastic responses in the [001]-oriented Fe_41_Ni_28_Co_17_Al_11.5_Ti_1.25_Nb_1.25_ (at.%) single crystals. Selecting single crystals avoids the grain boundary constraint in polycrystalline materials, which causes their brittle properties. The [001] orientation was selected because it provided the largest transformation or recoverable strain (8.7% in tension) based on theoretical calculations [6,7,8].

## 2. Materials and Methods

Iron, nickel, cobalt, aluminum, titanium, and niobium (99.9 wt%) were used as raw materials. Ingots of the FeNiCoAlTiNb alloy were melted from pure elements in a resistance furnace in a helium atmosphere. In order to obtain the homogeneous distribution of the elements in the bulk of the ingots, they were remelted three times. Fe_41_Ni_28_Co_17_Al_11.5_Ti_1.25_Nb_1.25_ (at.%) single crystals were grown using the Bridgman technique in a helium atmosphere on a Russian-made Redmet installation (Firm “Kristallooptika”, Tomsk, Russia) [28]. Dog-bone-shaped tensile samples with 1.5 mm × 3 mm × 8 mm gauge sections were cut from the single crystals with the tension axis along the [001] orientation. The single crystals were solution heat-treated at 1277 °C for 24 h and aged at 600 °C for 24, 48, and 72 h, designated as SXL600—24 h, SXL600—48 h, and SXL600—72 h.

Transmission electron microscopy (TEM) was conducted using a JEOL JEM−F200 electron microscope (Tokyo, Japan). The dark-field (DF) TEM images show precipitate distribution, and bright-field (BF) TEM images are used to observe precipitate size. A high-resolution TEM image is used to analyze precipitate composition and electron-dispersive X-ray spectroscopy (EDS) measurements. The crystal structures of the austenite matrix and precipitates are analyzed by examining the selected area electron diffraction (SAED) pattern. A focused ion beam (FIB) is used in TEM analysis sample preparation. Thermo-magnetic properties analysis was conducted using a superconducting quantum interference device (SQUID) magnetometer. The transformation temperatures, such as martensite start temperature (M_s_) and austenite finish temperature (A_f_), were characterized using SQUID. The magnetic fields are selected as 0.05 and 7 Tesla (T). The temperature range of the heating/cooling cycle was from 120 °C to −260 °C. The hardness results of [001]-oriented FeNiCoAlTiNb single crystals were characterized using a Vickers microhardness testing machine. The device was an FM-310 (FUTURE-TECH CORP, Kawasaki, Japan). Hardness values were measured on the {100} faces of solution-treated, SXL600—24 h, SXL600—48 h, and SXL600—72 h at room temperature

The tensile testing of superelasticity at room temperature was carried out on a universal tensile testing machine (AG-IS 50KN, Shimadzu, Kyoto, Japan). The strain rate was selected as 2 × 10^−4/^s. The strain during deformation was measured using the virtual optical extensometer with Vic-Gauge 2D version 7 software (Correlated Solutions, Irmo, SC, USA). Strain measurement using digital image correlation (DIC) can provide further insight into the local deformation. A speckle pattern was applied on the surface of a single-crystal sample for DIC analysis. During the tensile tests, sample deformation was recorded with a complementary metal–oxide–semiconductor (CMOS) at a frame rate of 5 Hz. VIC-2D software was employed to analyze the strain fields of the sample during the tests. The local strain fields were displayed in terms of normal strain parallel to the loading axis (ε_yy_) using the color bar.

## 3. Results and Discussions

### 3.1. Hardness of FeNiCoAlTiNb Single Crystals

**Figure 1:**
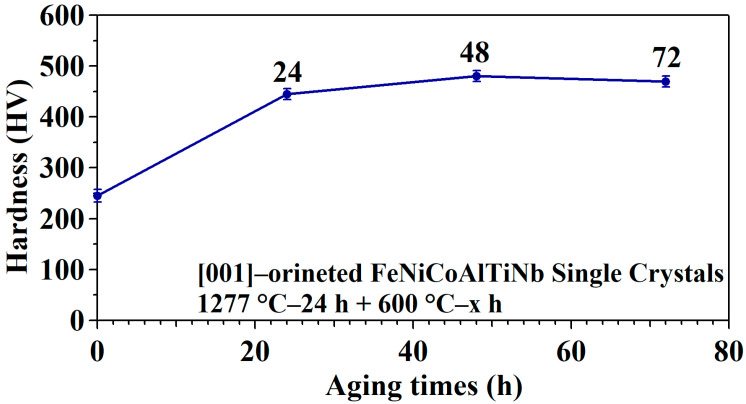
Hardness of [001]-oriented FeNiCoAlTiNb single crystals with different aging treatment durations.

Figure 1 shows the hardness of [001]-oriented FeNiCoAlTiNb single crystals after aging for different time durations. The hardness values of SXL600—24 h, SXL600—48 h, and SXL600—72 h are 445, 480, and 470 HV, respectively. High hardness was obtained when the aging time was 48 h. After reaching high hardness, the hardness began to decrease in value. The current hardness value (600 °C/48 h) is higher compared to 390 HV obtained in [001]-oriented FeNiCoAlTiNb single crystals (650 °C/6 h) [29]. For the [001]-oriented FeNiCoAlTa single crystals, Ma et al. reported that the highest hardness level (525 HV) was obtained for the aging condition (600 °C/90 h) [13].

### 3.2. Precipitate Morphology of FeNiCoAlTiNb Single Crystals

**Figure 2:**
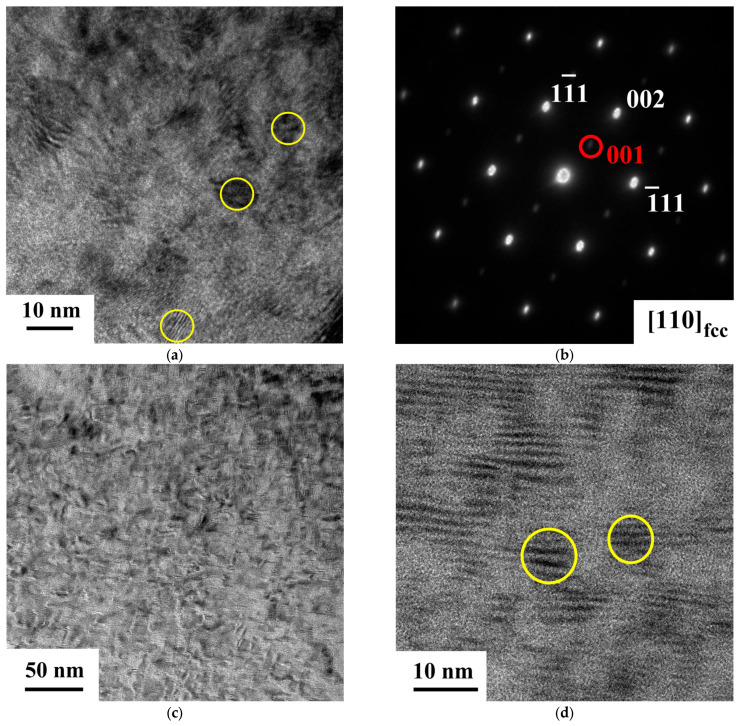

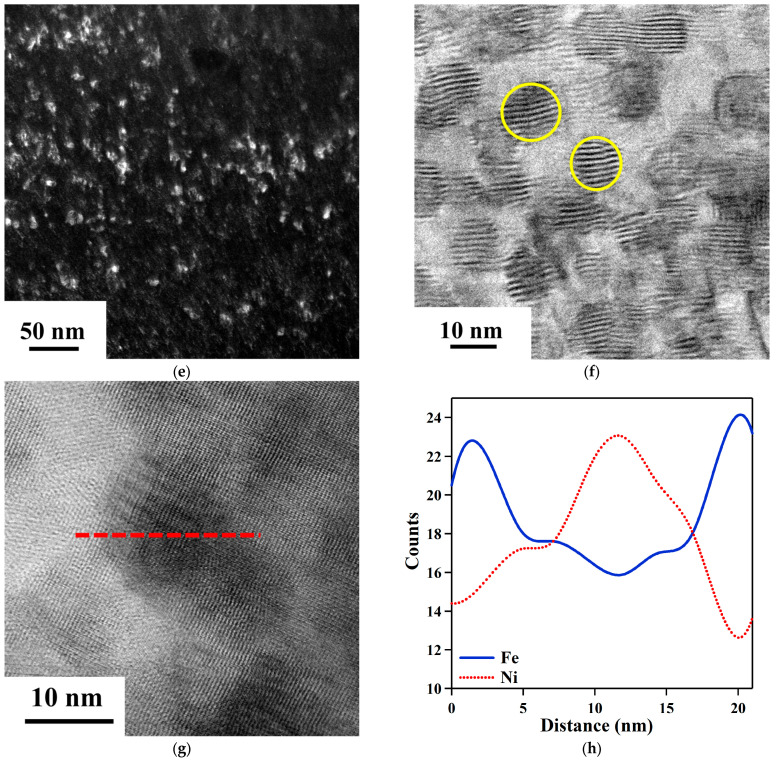
TEM analysis of [001]-oriented FeNiCoAlTiNb single crystals aging at 600 °C for different durations. (**a**) BF image for SXL600—24 h; the yellow circle indicates precipitate diameter. (**b**) Diffraction pattern results showing both austenite and precipitate structures. (**c**) BF image and (**d**) high-resolution TEM image for SXL600—48 h. (**e**) DF images for SXL600—72 h. (**f**) BF image for SXL600—72 h. (**g**) High-resolution TEM image of SXL600—72 h for the EDS line scan crossing one precipitate, as marked with a dashed line. (**h**) EDS line scan results showing the cps or count of Fe and Ni contents.

TEM observations were performed to reveal the precipitate morphology of the aged samples (SXL600—24 h, SXL600—48 h, and SXL600—72 h). Figure 2a presents the room temperature BF image of [001]-oriented FeNiCoAlTiNb SXL600—24 h. The yellow circle indicates the precipitate’s diameter. The average diameter of precipitates is 4~5 nm. The SAED pattern is taken from a 100-pole or [011]-zone axis. The results show that the austenitic matrix has an fcc structure, and the precipitate is an ordered L1_2_ structure, as shown in Figure 2b. Figure 2c shows the BF image for SXL600—48 h. The BF TEM image illustrates the precipitate distribution and shows a large number of precipitates. Figure 2d shows an HRTEM image and the precipitate with a size range from 8 to 9 nm. Figure 2e shows the DF TEM image of SXL600—72 h and reveals high precipitate density. The BF TEM image of SXL600—72 h shows that the average precipitate size is approximately 10~12 nm, as shown in Figure 2f. The precipitate size increases its diameter as the aging time increases. From Lauhoff et al. [29]’s TEM results, the precipitate size of FeNiCoAlTiNb aged at 650 °C for 6 h was reportedly around 6 nm. EDS line scans (dashed line) crossing precipitates were performed to evaluate changes in composition between the austenite matrix and precipitates, particularly the precipitates with high Ni content and Fe depletion. The observed L1_2_ precipitate with high Ni content in this study is similar to the FeNiCoAlTi results. Figure 2g shows an HRTEM of SXL600—72 h for composition analysis of precipitates and EDS line scans. In the figure, the dashed line represents the line analysis between the precipitate and the matrix. The line scan result is shown in Figure 2h. Because there are no significant differences between other elements (Co, Al, Ti, and Nb) in the region of the matrix and precipitate, the line scan result only shows the counts of Fe and Ni elements and excludes other elements. The line scan results show decreased Fe and increased Ni in the precipitate position. The composition analysis of precipitates is shown in Table 1. The result shows that the L12 precipitate has a high Ni content. Similar results were reported by Cassinerio et al. [30]. Based on the FeNiCoAlTi, FeNiCoAlTa and FeNiCoAlTaB results, the precipitates contain high Ni and low Fe contents [13,30,31].

### 3.3. Transformation Temperatures of FeNiCoAlTiNb Single Crystals

**Figure 3:**
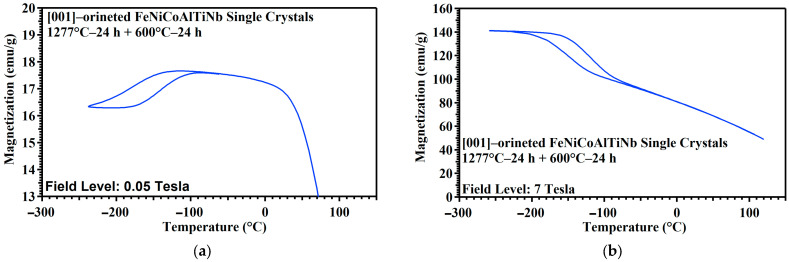

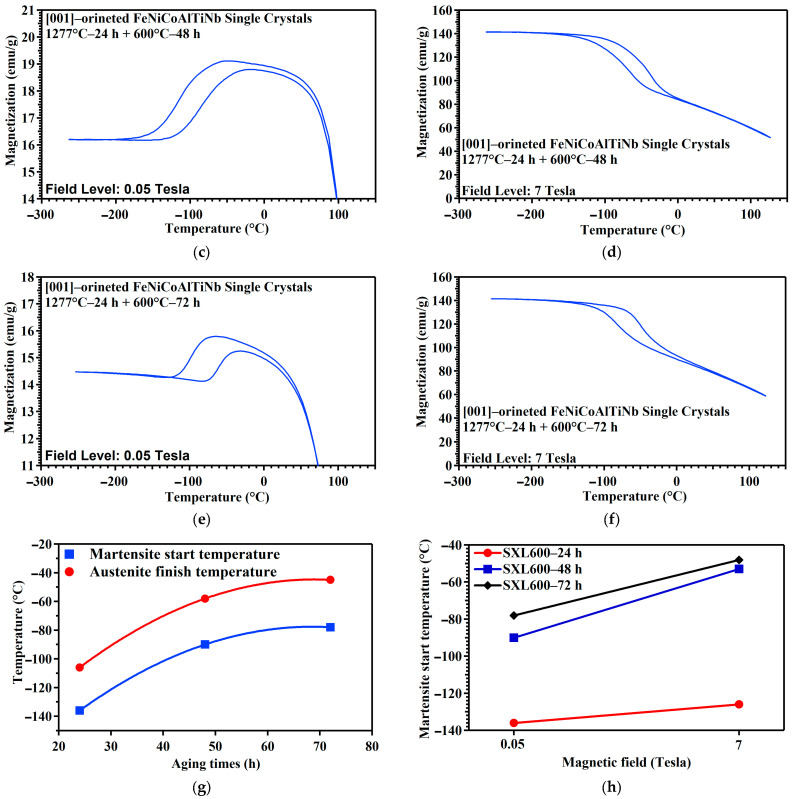
M−T curve for [001]-oriented FeNiCoAlTiNb single crystals under different aging conditions. Field level: 0.05 Tesla for (**a**) SXL600—24 h, (**c**) SXL600—48 h, and (**e**) SXL600—72 h. Field level: 7 Tesla for (**b**) SXL600—24 h, (**d**) SXL600—48 h, and (**f**) SXL600—72 h. (**g**) The relationship between transformation temperatures (A_f_ and M_s_) and aging time from the 0.05 Tesla measured results, and (**h**) M_s_ as a function of magnetic field for SXL600—24 h, SXL600—48 h, and SXL600—72 h.

Figure 3a–f show the thermo-magnetic M−T curve measured during the heating and cooling process under an external magnetic field of 0.05 and 7 T for the SXL600—24 h, SXL600—48 h, and SXL600—72 h across a temperature range of 120 °C to 260 °C. Based on this measurement, the martensitic transformation temperatures, i.e., the A_f_ and M_s_ values, determined using the tangent line method were 106 °C and 136 °C for SXL600—24 h, 56 °C and 88 °C for SXL600—48 h, and −45 °C and −78 °C for SXL600—72 h. The transformation temperatures (A_f_ and M_s_) values and temperature hysteresis of [001]-oriented FeNiCoAlTiNb single crystals under different aging condition are presented in Table 2. Figure 3g summarizes the transformation temperatures (A_f_ and M_s_) in three different aging conditions. The results show that transformation temperatures increase as aging time increases (24 to 72 h). From 48 to 72 h, the transformation temperatures reach saturated values. Increasing the transformation temperatures with increasing aging times is related to the composition change in the matrix. From the above EDS and line EDS scan results, the precipitates are high in Ni content, depleting Ni in the surrounding matrix. In several reference results, when the Ni content decreases in the austenitic matrix, the transformation temperature increases its value. Figure 3h summarizes the relationship between the transformation temperatures (M_s_) and the magnetic field. At a high magnetic field (7 Tesla), the M_s_ increases its value for all SXL600—24 h, SXL600—48 h, and SXL600—72 h. The result shows that the magnetic fields can induce the martensitic transformation temperatures for this material. The same trend can be found in the [001]-oriented FeNiCoAlTa single crystals [14].

According to the 0.05 Tesla magnetic field results, the martensite-phase magnetization is lower than that during the austenite phase. The magnetic values decrease as the aging times increase at low temperatures. At a high magnetic field (7 T), magnetization reached a maximum level of ~140 emu/g for SXL600—24 h during the cooling process. The maximum magnetization value for both SXL600—48 h and SXL600—72 h is the same as SXL600—24 h. It indicates that saturation magnetization is reached when the aging time is 24 h.

Moreover, the entropy of FeNiCoAlTiNb single crystals can be calculated using the Clausius–Clapeyron equation of the magnetic phase diagram [29] as follows:

(1) $$\Delta s\approx -(\frac{\Delta I}{\Delta T})\Delta H$$

where ΔT is the temperature hysteresis, ΔH is the magnetic field change, and ΔI indicates magnetization. For SXL600—24 h, when ΔH is 7 T, the magnetization is assumed to be −140 emu/g and ΔT is 30. The Δs is around 33 J/K^−1^kg^−1^.

### 3.4. Superelastic Responses of FeNiCoAlTiNb Single Crystals

**Figure 4:**
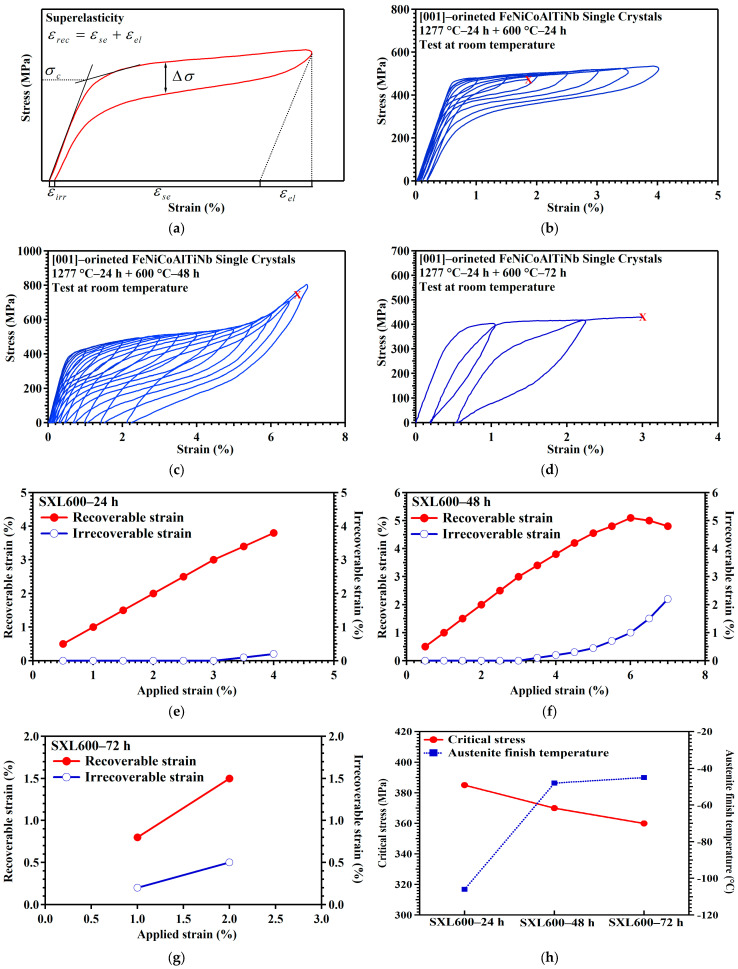
SE responses for [001]-oriented FeNiCoAlTiNb single crystals subjected to various aging heat treatments. (**a**) Illustration of the superelastic properties such as ε_rec_, ε_irr_, and σ_c_. Superelastic stress–strain curves for (**b**) SXL600—24 h, (**c**) SXL600—48 h, and (**d**) SXL600—72 h. X: point of failure. Summary of the recoverable and irrecoverable strain at various applied strain levels for (**e**) SXL600—24 h, (**f**) SXL600—48 h, and (**g**) SXL600—72 h. (**h**) Critical stress and austenite finish temperature for three aging conditions.

An illustration of how superelastic properties are obtained from superelasticity tests of [001]-oriented FeNiCoAlTiNb single crystals is presented in Figure 4a. In Figure 4a, ε_rec_ is the recoverable strain, which is the sum of ε_se_ (superelastic strain) and ε_el_ (elastic strain); ε_irr_ is the irrecoverable strain; and σ_c_ is the critical stress or critical transformation stress. Δσ refers to stress hysteresis, which is the width between the loading and unloading stress plateaus. The superelastic behaviors of SXL600—24 h, SXL600—48 h, and SXL600—72 h are shown in Figure 4b, 4c, and 4d, respectively. SXL600—24 h, SXL600—48 h, and SXL600—72 h fail during loading to 4.5%, 7.5%, and 3%, respectively. The fracture stress values are 474 MPa for SXL600—24 h, 749 MPa for SXL600—48 h, and 430 MPa for SXL600—72 h. The recoverable and irrecoverable strain of SXL600—24 h, SXL600—48 h, and SXL600—72 h are plotted against each applied strain, as shown in Figure 4e-g. Based on the results, the maximum recoverable strain is 3.8% for SXL600—24 h, 5.1% for SXL600—48 h, and 2% for SXL600—72 h. In addition, the critical stresses for SXL600—24 h, SXL600—48 h, and SXL600—72 h are 460, 370, and 360 MPa, respectively. The critical stress values of different aging conditions are related to transformation temperatures, especially the austenite finish temperature. Figure 4h summarizes the relationship between critical stress and austenite finish temperature. The austenite finish temperature values of −106 °C, −58 °C, and −45 °C for SXL600—24 h, SXL600—48 h, and SXL600—72 h, respectively, were determined from previous SQUID results. The result shows that the value of the critical stress decreases, and the value of the austenite finish temperature increases as the time increases from 24 to 72 h. In SMAs, the austenite phase is more stable at high temperatures. The austenite finish temperature of SXL600—72 h is higher than that of SXL600—24 h. As a result, less critical stress is required to induce the martensitic transformation for SXL600−72 h. The superelastic properties (critical stress, fracture stress, maximum recoverable strain, and ductility) of the samples under three aging conditions are summarized in Table 3.

### 3.5. Digital Image Correlation Results of FeNiCoAlTiNb Single Crystals

**Figure 5:**
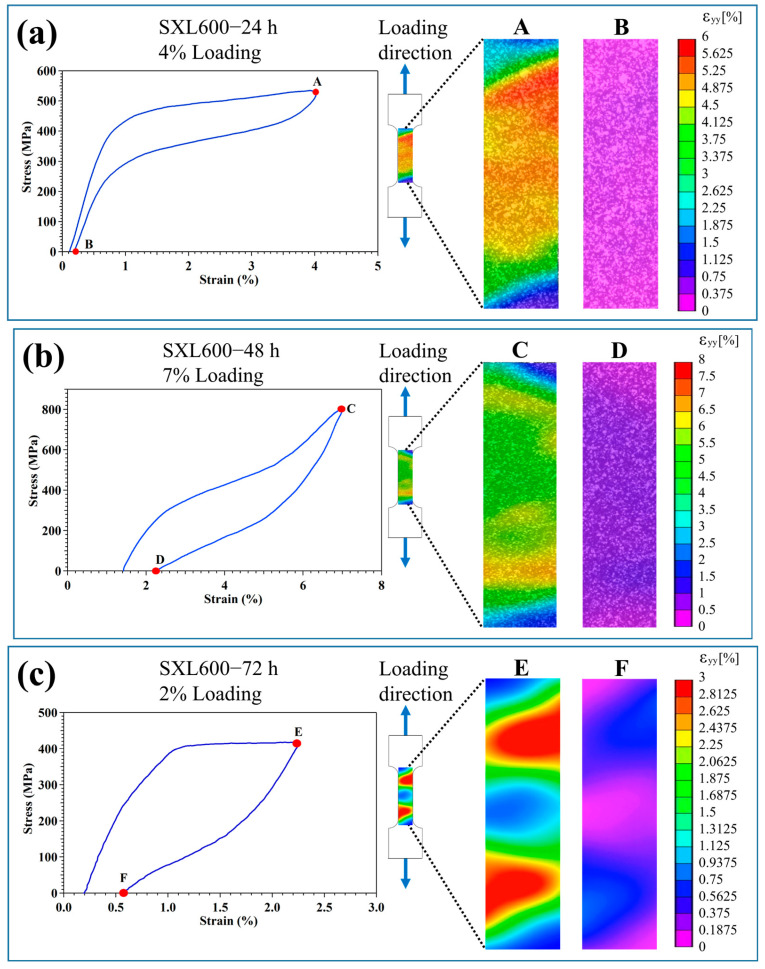
The strain fields of the [001]-oriented FeNiCoAlTiNb single crystals subjected to various aging heat treatments analyzed with the DIC method during the superelasticity test. (**a**) SXL600—24 h during the 4% loading, (**b**) SXL600—48 h during the 7% loading, and (**c**) SXL600—72 h during the 2% loading.

From the above superelastic results, SXL600—24 h shows small stress hysteresis and good recoverability, and the sample fails before showing pronounced irrecoverable strain. SXL600—48 h shows a maximum recoverable strain of up to 5.1%; however, the obvious accumulation of irrecoverable strain was observed after applying 5% strain. SXL600—72 h shows small recoverable strain and poor recoverability. To further understand the deformation mechanism during the superelasticity test, the DIC method was performed on the previous cycle before sample fracture to observe the strain distribution during the test. Figure 5a–c show the DIC results for SXL600—24 h, SXL600—48 h, and SXL600—72 h during the 4%, 7%, and 2% loading, respectively. Based on the Bain distortion for FCC-BCT transformation, two martensite variants are in the tensile direction. In the FeNiCoAlTa and FeNiCoAlTi results, they observed at least two different martensite variants during the superelasticity test [15,22]. Two variants can form corresponding variant pairs to assist the deformation during the superelasticity test. As a result, SXL600—24 h and SXL600—48 h show good superelastic behavior. Based on the reference, the twin planes of martensite variants belong to {110} planes [22]. The superelastic result of SXL600—24 h shows the plateau region and small recoverable strain before failure. Almost no residual martensite was observed based on the DIC strain contour plot at point B (Figure 5a) after 4% loading. When the applied strain increases to 4.5%, the sample fails before showing a large irrecoverable strain. It is related to the martensite variant–variant interaction growing intense during the superelasticity test. In the beginning cycle of the superelasticity test, martensite phases can transform into austenite phases. When the applied strain level continuously increases, it reduces the volume fraction of untransformed austenite phases. As a result, increasing the variant–variant interactions leads to early fracture [13,14]. For SXL600−48 h (the peak aging conditions), the precipitates increase the strength of the matrix to resist the fracture stress and increase the recoverable strain values. Although SXL600—48 h shows the maximum recoverable strain up to 5.1%, the sample also shows cyclic degradation at room temperature, especially after 6% loading. For the cyclic stability of FeNiCoAlTiNb alloys, the cyclic functional degradation behavior is strongly related to test temperatures [29]. Krooß et al. [15] and Lauhoff et al. [29] reported that the cyclic stability decreases with increasing test temperature. The single crystal shows good cyclic stability at a test temperature of −130 °C. When the test temperature is at room temperature, cyclic stability drastically decreases. Cyclic degradation leads to dislocation formation. They pin the martensite phase and lead to residual martensite staying in the sample after the test, as shown in the DIC strain contour plot at point D (Figure 5b). The residual martensite was attributed to the irrecoverable strain. For the DIC result of SXL600—72 h, a large local stress field was generated in the samples. The deformation is dominated by plastic slip on the {111} slip planes [22]. Consequently, the dislocations are generated during the test. Dislocations act to pin the martensite phase boundary to prevent the reverse transformation to austenite. The martensite is residual in the sample after unloading, shown in the DIC strain contour plot at point E (Figure 5c), causing poor recoverability for SXL600—72 h.

**Figure 6:**
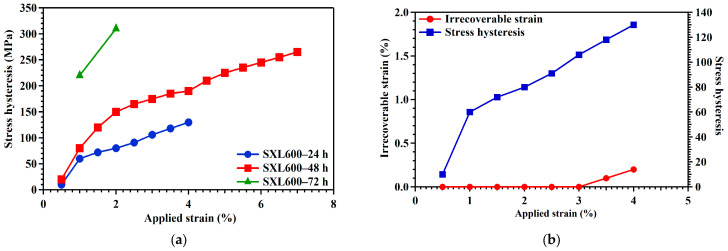

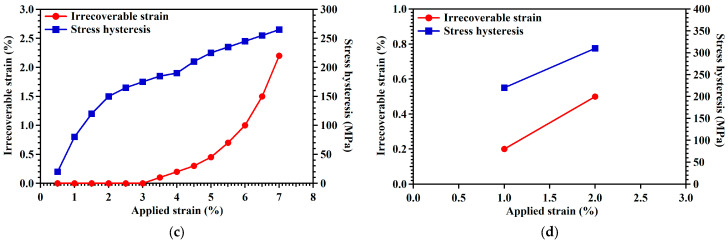
(**a**) Stress hysteresis as a function of applied strain in three different aging conditions. The irrecoverable stain and stress hysteresis of the applied strain for (**b**) SXL600—24 h, (**c**) SXL600—48 h, and (**d**) SXL600—72 h. Data points were extracted from the superelastic results, as shown in Figure 4.

The precipitate morphology can tailor the superelastic properties such as stress hysteresis and recoverable and irrecoverable strains. For the FeNiCoAl-based system, the different aging heat treatment times affect the precipitate size, superelastic responses, and functional or cyclic stability [14,15]. Stress hysteresis is related to energy dissipation. High stress hysteresis values are related to high frictional resistance to interfacial motion and high stored elastic strain energy dissipation. Large stress hysteresis indicates that the dislocations are easily generated during stress-induced martensitic transformation [32]. Figure 6a summarizes the stress hysteresis as an applied strain function for SXL600—24 h, SXL600—48 h, and SXL600—72 h. Stress hysteresis values are shown in Figure 4. Stress hysteresis increased as the applied strain increased. At the 1% applied strain level, SXL600—72 h stress hysteresis was higher than SXL600—24 h. Superelasticity stress hysteresis depends on precipitate coherency. Stress hysteresis is low in both SXL600—24 h and SXL600—48 h because the precipitate is coherent with the matrix during the superelasticity test. When the precipitate size is large for SXL600—72 h, precipitations act as obstacles to reducing the mobility of austenite and martensite boundaries. Frictional resistance to phase boundary motion increases and amplifies the hysteresis loop. On the other hand, with further increases in precipitate size, SXL600—72 h may result in a loss of coherency and reduced effects of precipitation hardening. Additionally, with increasing aging time, increases in precipitate size and stress hysteresis width result from decreases in the recoverable strain and cyclic stability. Figure 6b–d summarize the irrecoverable stain and stress hysteresis of the applied strain for SXL600—24 h, SXL600—48 h, and SXL600—72 h. For SXL600—24 h, the precipitate is 4–5 nm and coherent with the matrix, resulting in small stress hysteresis, indicating that the small irrecoverable strain and low frictional resistance to interfacial motion were observed during the 4% strain test. For SXL600—48 h, the precipitate size is 8~9 nm and still coherent with the matrix. The pronounced stress hysteresis value is increased to over 200 MPa, and the stress–strain curve shows the irrecoverable strain after 5% loading. The increasing irrecoverable strain is due to cyclic degradation in superelasticity. Cyclic degradation causes residual martensite. In the subsequent loading, residual martensite accumulation leads to increased irrecoverable strains. Moreover, SXL600—48 h cyclic degradation is more obvious than SXL600—24 h and is related to precipitate size. Based on the FeNiCoAlTa results [14,15], increased aging times not only increase the precipitate size but also amplify cyclic degradation. For SXL600—72 h, the precipitate size is 10~12 nm, and precipitations lose coherency, resulting in dislocation formation and reduced austenite and martensite mobility. On the other hand, SXL600—72 h recoverability decreases, and the precipitates will join together and become thicker due to more diffusion. The distance between precipitations increases stress fields and dislocation formation. The dislocations enlarge the stress hysteresis width and stabilize martensite, causing poor recoverability.

## 4. Conclusions

The main findings of this study on the effect of aging on the superelastic properties of [001]-oriented FeNiCoAlTiNb single crystals under tension can be summarized as follows:

1. TEM analysis revealed that the nano-precipitate crystal structure is L1_2_. The precipitate size increases its value (4 nm to 12 nm) when the aging heat treatment time increases from 24 to 72 h. EDS line scan results show that the nickel content in the precipitate region increases with increasing aging time while the iron content decreases. The precipitates are rich in Ni content.
2. Thermo-magnetization measurement results show that the transformation temperatures increase as the aging times increase. From 48 to 72 h, the transformation temperatures reached saturated values. Applying a high magnetic field of 7 T, the transformation temperatures rose, indicating that the magnetic induction of the martensitic transformation temperatures can be used in magnetic applications in the future.
3. The superelasticity test results show the maximum recoverable strain is 3.8%, 5.1%, and 2% for SXL600—24 h, SXL600—48 h, and SXL600—72 h, respectively. The critical stresses are 460 MPa for SXL600—24 h, 370 MPa for SXL600—48 h, and 360 MPa for SXL600—72 h.
4. The DIC results show that two martensite variants can activate in the tensile deformation. The precipitate size is coherent within the matrix for SXL600−24 h and SXL600—48 h. SXL600−24 h fails when applying the 4.5% strain due to the martensite variant–variant interaction becoming intense, leading to early fracture. The SXL600—48 h shows the maximum recoverable strain up to 5.1%; however, pronounced cyclic instability was observed after applying the 5% strain. The poor functional ability causes residual martensite and results in decreased abilities. A large size was observed for SXL600—72 h. Precipitations act as obstacles to reduce the mobility of austenite and martensite boundaries. Frictional resistance to phase boundary motion increases and amplifies the stress hysteresis loop. Dislocations act to pin the martensite phases to prevent them from reversing back to austenite, resulting in poor recoverability.

## Figures and Tables

**Table 1:** EDS analysis of the precipitates for SXL600—72 h.

	Al (at%)	Nb (at%)	Ti (at%)	Fe (at%)	Co (at%)	Ni (at%)
Measured	7.9	1.0	1.2	37.8	19.3	32.7
Nominal	11.5	1.25	1.25	41	17	28

**Table 2:** Transformation temperatures and temperature hysteresis, as determined from SQUID measurements for SXL600—24 h, SXL600—48 h, and SXL600—72 h.

Aging Conditions	Magnetic Field (T)	M_s_ (°C)	A_f_ (°C)	Temperature Hysteresis (°C)
SXL600—24 h	0.05	−136	−106	30
	7	−126	−96	30
SXL600—48 h	0.05	−90	−58	32
	7	−53	−21	32
SXL600—72 h	0.05	−78	−45	33
	7	−48	−15	33

**Table 3:** Superelastic properties of FeNiCoAlTiNb single crystals under different aging conditions.

Superelastic Properties	SXL600—24 h	SXL600—48 h	SXL600—72 h
Critical stress (MPa)	460	390	370
Fracture stress (MPa)	472	750	425
Maximum recoverable strain (%)	3.8	5.1	2
Ductility (%)	4	7	1.5

## Data Availability

The original contributions presented in this study are included in the article; further inquiries can be directed to the corresponding author.
